# Attitudes, preference and personality in relation to behavioral intention of autonomous vehicle use: An SEM analysis

**DOI:** 10.1371/journal.pone.0262899

**Published:** 2023-02-13

**Authors:** Ling Ding, Xu Yang

**Affiliations:** 1 College of Transportation Engineering, Chang’an University, Xi’an, Shaanxi, China; 2 School of Highway, Chang’an University, Xi’an, Shaanxi, China; Bucharest University of Economic Studies, ROMANIA

## Abstract

Autonomous vehicles (AVs) are entering the market, which will have a great impact on future decision making on mode choice in transportation systems. The aim of this study is to explore the determinants which influence travelers’ intentions to use AVs based on structural equation modelling (SEM). 310 valid sets of data from an online survey were collected to analyze factors which influence travelers’ intentions. Data analyses were conducted using IBM SPSS Statistics 23 and AMOS 23. The results showed that personality and preferences in relation to AVs are the main potential factors that cause travelers’ AVs use. Attitudes to modal services also affect intentions to use AVs. Personality has a significant positive effect on both attitude and preferences. The results provide exploratory empirical support for all hypotheses. The research results will help understand travelers’ choice motivation from psychological and service perspectives, and provide support for governments and enterprises to improve the management and services of autonomous vehicles.

## 1 Introduction

The choice of travel mode is a complex decision-making process, which is affected by many factors, such as individuals’ social characteristics, travel characteristics, and the service of different modes [1]. Individuals’ social characteristics include gender, education and occupation. Modal attributes and individual social characteristics are usually used in most models of travel mode choice to explain passengers’ choices. Travelers’ psychological factors are also important for travel behaviors [2, 3]. In recent years, the effects of psychological factors on travel behavior have received increasing attention in order to improve understanding of travel behavior. It is believed that the psychological factors that affect individual mode choice include attitudes, perceptions and norms [4–6]. Usually perceived attitudes are latent variables, which cannot be observed directly, but need to be measured by relevant questions. For example, different respondents may have different expectations of the metro service level. Some people may expect a higher level of comfort than economy, while others expect a higher level of safety than convenience. In the empirical studies on travel mode choice, psychological factors are better predictors of mode choice decisions than modal differences and individual social characteristics [7]. Researchers address these psychological factors as unobservable, or latent variables in mode choice models to gain insights into individuals’ processes of mode choice decision-making [8–10]. These studies provide insights into the relationship between psychological factors and travel behavior. Empirical findings indicate that the addition of psychological factors to mode choice models improves predictions of travel behavior. For example, a model with added psychological factors demonstrated greater explanatory and predictive power than a model having only sociodemographic and modal variables [11]. In models, travel-related attitudes explain the variation of mode choice behavior better than sociodemographic variables [12, 13].

Attitude, a critical factor in explaining human behavior, refers to the potential tendency to have favorable (or unfavorable) reactions to psychological objects [14]. An individual’s attitude to a mode of transport (the performance of the mode) influences his/her intention to make a mode choice decision. According to the theory of planned behavior [15], an individual’s attitude to a behavior is the sum of his or her beliefs about the behavior (for example, it is convenient to travel by car) multiplied by his or her emphasis on each belief. Although it is recognized that attitudes affect mode choice, in most researches, the variable of attitudes is added to the travel utility function to study the impact of attitudes on mode choice.

Autonomous vehicles are expected to reduce traffic accidents caused by drivers [16, 17]. Litman [18] expects that AVs’ beneficial impacts on safety and congestion are likely to appear between 2040 and 2060. Past studies tend to adopt a stated preference (SP) approach to understand travelers’ attitudes to AVs and quantify the intention to use AVs. Schoettle and Sivak [19] conducted a survey in the U.K., the U.S., and Australia to understand respondents’ perceptions of AVs. Many factors (such as age, gender, income, education level) have been investigated to examine their influence on the intention to use AV [20–22]. Other researchers have suggested that consideration of psychological factors may facilitate the understanding of people’s mode choice intention [23, 24]. Gefen [25] considered that trust and risk would affect attractiveness and have an impact on intentions to use technology. As a variable to simple assignments of values, the importance of attitudes in travel mode choices was always the focus in previous studies [26, 27]. However, mechanisms affecting attitudes have been much less studied, in particular, whether the attitude is correlated to sociodemographic characteristics (e.g. income, occupation). This paper extends the literature on the intention to use AVs by providing insights into the psychological factors and mechanisms affecting attitudes to them. Previous researches indicate that economic, pragmatic and social factors are typically influential [28]. The present study focuses exclusively on personality, attitude, and trust as predictors of intention to use AVs. We seek to examine the mechanism of the formation of subjective attitudes and the predictive validity of latent variables (attitude and preference) in the case of predicting future intention to use AVs.

The rest of the paper is structured as follows: Section 2 provides the theoretical methodology which outlines the framework of attitudes to mode choice behavior and the determinants of attitude formation. An established theoretical model which proposes antecedents to attitudes to transport mode choice is presented as a basis for investigation. In Section 3 we describe the survey approach and the questionnaire. Individual, household and travel characteristics of the sample are also analyzed. Section 4 is the results and discussion part. The paper concludes by providing a summary of the findings, its limitations, implications for policy and suggestions for further research.

## 2 Methodology

### 2.1 SEM model

Whereas previous mode choice models have included convenience and comfort of modes [10, 29], we extend the list of latent variables including environmental preferences and individual preferences such factors as accessibility, flexibility, predictability, and reliability. A structural equation model (SEM) is developed to explain the effects of different factors on mode choice. The framework for modelling over two stages is based on previous studies [2, 30]. SEM is a flexible linear-in-parameters model to deal with latent variables and complex correlations, and has been widely used in research on travel decision-making. SEM combines factor analysis and simultaneous equation models to represent the relationship between observed variables and latent variables. A SEM model consists of a measurement model and a structural model. The measurement model defines and confirms the latent variables, while the structural model explores the relationships between the variables.

**Fig 1:**
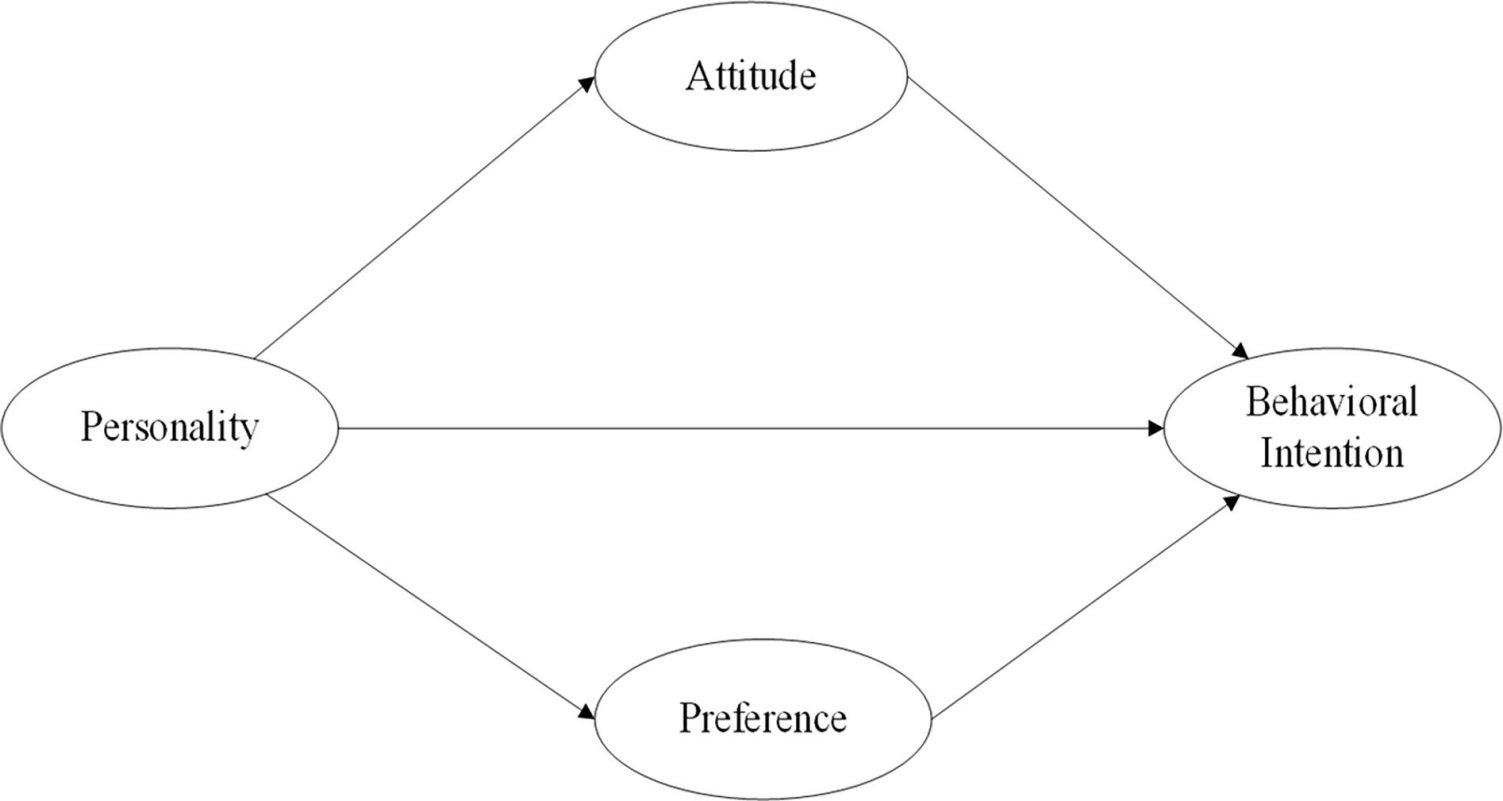
Modelling framework (personality-attitude & preference-intention (PAPI)).

Firstly, we use confirmatory factor analysis (CFA) to assess the reliability and validity of the measurement models. Secondly, we test the relationship between factors and mode choice using a structural model. In this study, the behavior, which is treated as an indirectly observed measure, reflects the behavioral intention in relation to AVs. Fig 1 gives the framework for modeling. We employed the Chi-square, Chi-square to degree of freedom ratio, Comparative Fit Index (CFI), Goodness of Fit Index (GFI), Incremental Fit Index (IFI), Root Mean Square Error of Approximation (RMSEA) and the Tucker-Lewis Index (TLI) to evaluate the fit of the hypothesized models to the empirical data [31, 32].

To sum up, we assume that passengers do not always make decisions rationally and unilaterally according to mode attributes such as actual waiting time and cost. Therefore, we propose the following hypotheses:

**Hypothesis 1.** Personality relates to household income, academic level, occupation and car ownership.

**Hypothesis 2.** Attitude relates to interior environment (IE), rest (RE), accessibility (AC), flexibility (FL), predictability (PR), reliability (AOT), and environmental preference (EP).

**Hypothesis 3.** Preference relates to safety (SE), comfort (CO), convenience (COV), and responsibility (IAL).

**Hypothesis 4.** Personality relates to attitude and preference.

**Hypothesis 5.** Personality and attitude to mode choice have effects on intention to use AVs.

**Hypothesis 6.** Trust (preference) has positive influence on the use of AVs.

A SEM model (Fig 1) considers attitudes when travelers make mode choice decisions, attitudes to AVs, and sociodemographic variables. The importance of latent variables when a traveler chooses a mode can be ranked from irrelevant to very important. Next, what aspect of sociodemographic characteristics affect travelers’ attitude when they make a mode choice decision? We need to quantify the relationship among personality, attitudes and behavioral intention. The stronger the behavior intention, coupled with the requisite opportunities and resources, the more probable behavior [15].

### 2.2 Variables of attitudes

A structural equation model consists of measurement models and structural models. Measurement models connect the observable variables with the latent variables, and the structural models connect the latent variables with each other using systems of simultaneous equations. Latent variables are phenomena which are considered to exist but cannot be observed directly. Variables of subjective attitudes in this study include personal attitudes when making a mode choice decision and attitudes to AVs. The personality of people includes their age, gender, household income, academic level, and occupation. Detailed descriptions of attitudes are shown in Table 1.

**Table 1:** Description of attitudes and preference variables.

	Variables	Description
Attitude	Interior environment (IE)	The importance of the interior environment to mode choice
	Rest (RE)	The importance of whether we can have a rest during the trip
	Accessibility (AC)	The importance of direct access to the destination
	Flexibility (FL)	The importance of whether to wait for the vehicle
	Predictability (PR)	Vehicle arrival time can be predicted.
	Reliability (AOT)	The importance of being able to arrive at the destination on time.
	Environmental preference (EP)	The importance of environmentally friendly travel mode
Trust (Preference)	Safety (SE)	Extent the safety of AVs is trusted.
	Comfort (CO)	Extent to which the comfort of AVs is trusted.
	Convenience (COV)	Extent to which the convenience of AVs is trusted.
	Responsibility (IAL)	Extent to which the identification of accident liability of AVs is trusted.

Attitudes to each aspect when making a mode choice decision were assessed. Unless indicated otherwise, items were scored from 1 to 5, with higher scores denoting greater importance of the variable when choosing a transport mode. For example, the statement “I believe that the mode enables me to arrive at the destination on time” is rated on a scale from the unimportant (1) to the most important (5) [17]. Following the indirect measurement approach [33], we assessed the extent to which the four primary beliefs identified how people trust these beliefs, and measured a 10-point semantic scale ranging from “very mistrustful” to “very trustful” (10).

Participants’ behavioral intentions to use AVs were assessed by three items (Questions 1–3) based on previous studies [33, 34]. Responses were measured by a unipolar 10-point Likert scale with response categories ranging from “strongly disagree” [17] to “strongly agree” (10). The items assessed self-prediction (“In the future, I expect to use AVs”), desire (“In the future, I want to use AVs”) and intention (“In the future, I intend to use AVs”). The scores ranged from 3 to 30 with a high score indicating a high intention to use AVs for trips.

## 3 Data collection

### 3.1 Description of experiment

In order to better understand passengers’ behavioral intentions in relation to AV use, we invited an adult population (≥18 years) to complete an online questionnaire. The questionnaire consisted of two main parts administered using an online approach: ‘SoJump’ (https://www.sojump.cn/), a professional online survey platform which allows users to specify questions, store answers and process data. Individuals were asked to rate the importance (on a 5-point scale) of certain attributes (for example, “Reliability is very important when I choose a transport mode”). This enabled us to explore different importance of factors in terms of mode choice-related attitudes. In addition to questions on socioeconomic issues and behavioral intentions, the survey also contained attitudinal questions to measure potential (latent) variables, assuming that latent variables are important choice.

The questionnaire is composed of four parts: the first part contains questions related to the sociodemographic characteristics of the travelers. The second part is designed to collect the travelers’ attitudes to different services when making a mode choice decision. We developed measurement indicators for the seven attitude dimensions: interior environment, rest, accessibility, flexibility, predictability, reliability, and environmental preference. Respondents were asked to rate attitudinal questions relating to these seven dimensions on five-point Likert-scales ranging from “irrelevant” (not important at all) to “very important”. The third section includes questions about the extent to which they trust AVs (preference for AVs). Respondents were asked to rate four measures: safety, comfort, convenience and responsibility. The survey ends with a section where we measure respondents’ future intentions: “In the future I expect to use AVs”; “In the future, I want to use AVs”; and “In the future, I intend to use AVs”. Respondents were asked to indicate the extent to which they agree with each statement.

A total of 426 questionnaires were collected. After eliminating incomplete surveys and ineligible participants, 310 eligible questionnaires (73%) were collected for analysis. The respondents were distributed in Beijing, Shanghai, Jiangsu, Henan and Sichuan. Overall, the demographic mix is considered to be the representative of potential AVs users in China. In our analysis, we used 310 useable responses, which is much higher than the minimum required to reduce deviation to an acceptable level for SEM calculation [35, 36].

### 3.2 Data analysis

Descriptive analysis was employed to describe the respondents’ background characteristics (e.g., basic sociodemographic variables). With regard to the association with attitudes when making a mode choice decision, individuals’ personal characteristics are further examined. Such descriptive analyses as frequencies, percentages, means and standard deviations, were calculated. Following our personality-attitude & preference-intention [37] framework, the collected data were analyzed using SEM. However, all statistical analyses were conducted using SPSS 23.0.

A summary of the individual and household characteristics of the respondents is presented in Table 2. 49.7% of the sample is female. More than 50% of the respondents are aged between 26 and 35 years old, and about 69% of the sample households have a personal income between RMB 50,000 and 300,000 per year. Of our sample, 246 respondents (79.4%) have bachelor’s degree or above. The increasing number of people using smartphones has resulted in easier access to online surveys. According to Bruijne and Wijnant [38], those who have higher educational qualifications are more likely to use smartphones to complete online questionnaires, and similar results were reported for younger respondents. In other words, younger respondents or those who have high educational qualifications are more willing to participate in online surveys.

**Table 2:** Sample information (N = 310).

Individual level	Categories	Number	Percentage (%)
Age group (AG)	18–35	183	59.0
	36–55	105	33.9
	56–65	21	6.8
	>65	1	0.3
Gender (GED)	Female	136	49.7
	Male	167	50.3
Academic level (AL)	High school diploma or below	64	20.6
	Bachelor degree	211	68.1
	Master or higher degree	35	11.3
Occupation (OC)	Student	13	4.2
	State-owned enterprise or institution	109	35.2
	Government agency	15	4.8
	Private company	148	47.7
	Self-employed	25	8.1
Household income (HI)	Salary less than ¥20,000 p.a.	16	5.2
	Salary between ¥20,000 and ¥50,000 p.a.	55	17.7
	Between ¥50,000 and ¥150,000 p.a,	128	41.3
	Between ¥150,000 and ¥300,000 p.a.	86	27.7
	¥300,000 or more p.a.	25	8.1
Car Ownership	0	77	24.8
	1	197	63.5
	2	34	11.0
	>2	2	0.6

Table 3 reports the means and standard deviations (SDs) of all measures used in the model estimation. As mentioned above, we used these attitude items as indirect measure of attitudes to the use of AVs. The item means and SDs show the strength of belief of the respondents in relation to mode choice and AVs. Regarding person-related beliefs (mode choice attitude), we found that these beliefs were often extremely important. In specific terms, the belief “preference for accessibility” was the most important (mean = 8.42) of the 7 attitude indicators. However, the belief “rest in the vehicle” was the least important (Mean = 5.80) of the mode choice attitude indicators.

**Table 3:** Means and standard deviations for attitude measures and intentions.

Measure	Item	Mean	SD
Measure of attitude	The importance of the interior environment for mode choice (IE)	7.94	1.84
	The importance of whether we can have a rest or read books during the trip (RE)	5.80	2.00
	The importance of direct access to the destination (AC)	8.42	1.78
	The importance of needing to wait for the vehicle (FL)	7.76	1.8
	Vehicle arrival time can be predicted (PR)	7.86	2.02
	Ability to arrive at the destination on time (AOT)	8.26	2.1
	The travel mode is environmentally friendly (EP)	7.10	2.2
Measure of trust (preference)	I trust the safety of AVs (SE)	7.19	2.18
	I trust the comfort of AVs (CO)	8.07	1.86
	I trust the convenience of AVs (COV)	8.10	1.93
	I trust the identification of accident liability of AVs (IAL)	6.64	2.30
Intention	In the future, I expect to use AVs (Q1)	4.90	2.02
	In the future, I want to use AVs (Q2)	6.21	2.10
	In the future, I intend to use AVs (Q3)	7.26	2.15

In relation to attitudes to AVs, “convenience of AVs” was the most important indicator of AV preference (Mean = 8.10) and “identification of accident liability” was the least important (Mean = 6.64). As Table 3 shows, the Cronbach alpha coefficients range from 0.79 to 0.81. All coefficients of Cronbach alpha are greater than the threshold value of 0.6, which indicates that the measures captured what they were supposed to measure (internal consistency) [33]. From both theoretical and empirical perspectives, personality (defined as individuals’ social characteristics) has an impact on behavioral intentions (i.e. future intentions to use AVs), via attitudes (in this case, attitudes about the importance of modal attributes on mode choice) and preferences (i.e. trust in AVs).

## 4 Results and discussion

### 4.1 Results

**Fig 2:**
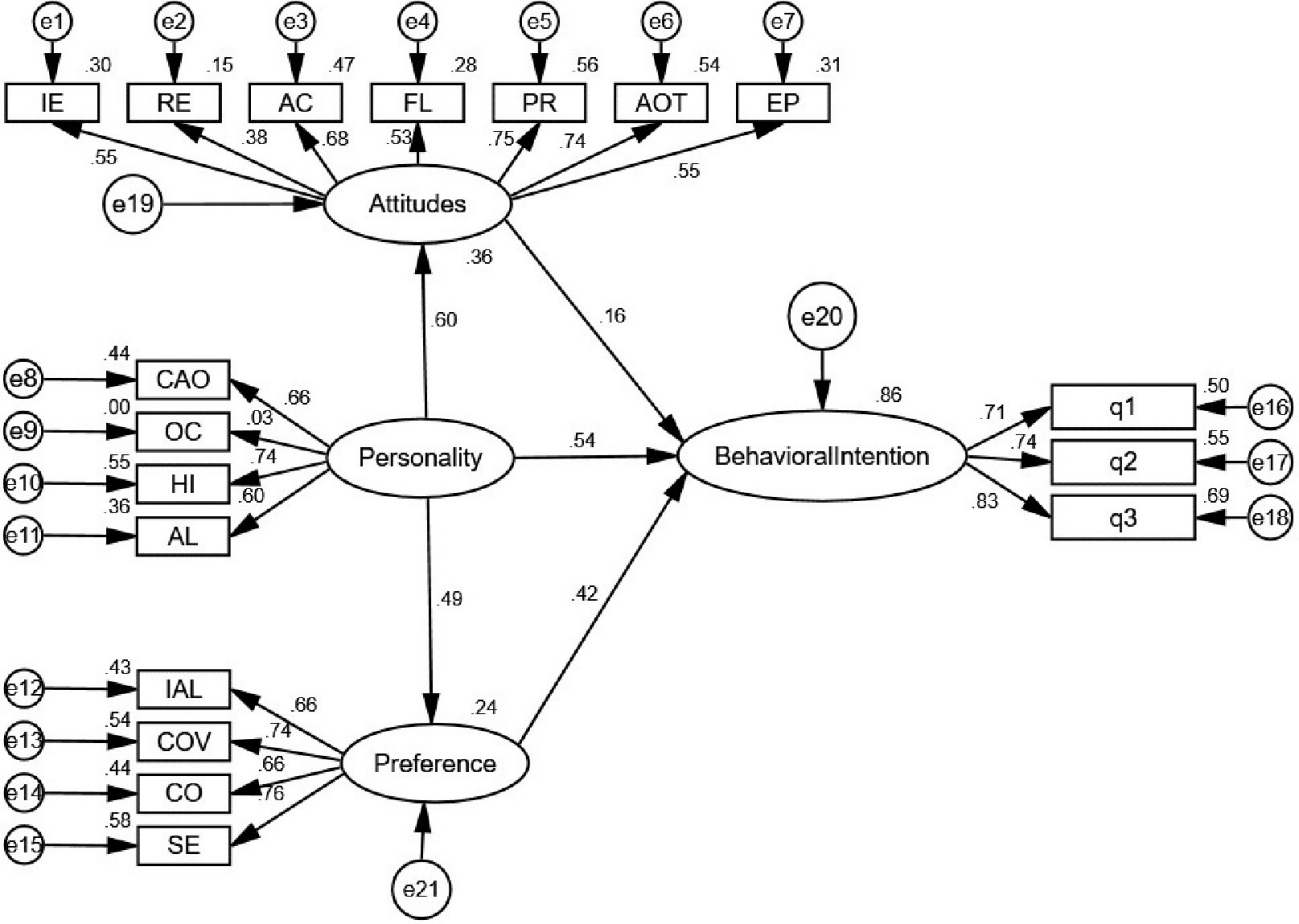
Structural equation diagram for analysis of intention to use AVs.

The SEMs in Fig 2 were estimated using AMOS 23.0. Table 4 reports the results for both the measurement and structural models. The Chi-square for the SEM is 300.523 (df = 130, χ^2^/df = 2.312, RMSEA = 0.045, GFI = 0.893, AGFI = 0.860, NFI = 0.855, CFI = 0.911, IFI = 0.912, TLI = 0.895). The Incremental Fit Index (IFI), Comparative Fit Index (CFI) and Tucker-Lewis Index (TLI) values are equal to or greater than 0.90 [31, 32], the χ^2^/df value is between 1 and 3 [39], and the RMSEA values are less than 0.08 [40, 41], indicating that the SEM fits the data well. A graphical representation of Table 4 is shown in Fig 2. Table 6 shows the standardized direct, indirect and total effects between the latent variables.

**Table 4:** Results of model path coefficients.

Relation		Coeff.	S.E.	C.R.	P	Std. Coeff.
Structural model						
Attitude ←	Personality	0.77	0.12	6.30	***	0.60
Preference ←	Personality	1.86	0.31	5.90	***	0.49
Intention ←	Personality	1.92	0.35	5.75	***	0.54
Intention ←	Attitude	0.43	0.19	2.29	0.02	0.15
Intention ←	Preference	0.39	0.06	6.21	***	0.42
Measurement model						
CAO ←	Personality	1.00	—	—		0.66
HI ←	Personality	1.81	0.18	10.06	***	0.74
OC ←	Personality	0.09	0.18	0.50	0.62	0.03
AL ←	Personality	0.83	0.09	8.61	***	0.59
IE ←	Attitude	1.00	—	—		0.55
RE ←	Attitude	0.75	0.13	5.63	***	0.38
AC ←	Attitude	1.19	0.14	8.48	***	0.68
FL ←	Attitude	0.93	0.13	7.17	***	0.52
PR ←	Attitude	1.54	0.17	8.90	***	0.74
AOT ←	Attitude	1.52	0.17	8.84	***	0.73
EP ←	Attitude	1.19	0.16	7.42	***	0.55
IAL ←	Preference	1.00	—	—		0.66
CO ←	Preference	0.81	0.08	9.61	***	0.66
COV ←	Preference	0.94	0.09	10.39	***	0.73
SE ←	Preference	1.09	0.10	10.59	***	0.76
q1 ←	Intention	1.00	—	—	—	0.70
q2 ←	Intention	1.09	0.09	11.94	***	0.74
q3 ←	Intention	1.25	0.09	13.13	***	0.83

#### 4.1.1 Measurement model

In order to examine the reliability and validity of constructs in the model, an evaluation of the measurement model was conducted. With the exception of the variables of occupation and rest, the standardized factor loadings (SFLs) (item reliability) are statistically significant (<0.001) and above the minimum criterion of 0.50 (ranging between 0.53 and 0.83) [42]. The results of the analysis of Cronbach’s alpha reliability show that the coefficient values for attitude, preference and behavior intention are 0.80, 0.79, and 0.81, respectively. Furthermore, confirmatory factor analysis (CFA), including structural reliability (CR) and average variance extraction (AVE), were used to test the reliability and validity of the measurement instrument. The factor loadings extracted from the model were used to calculate composite reliability. AVE measures the internal consistency of a construct by calculating the variance of latent variables captured from its measurement items relative to the variance caused by measurement errors. Table 5 indicates that the construct reliability (CR) estimates range from 0.75 to 0.87, exceeding the threshold value of 0.7 [42]. All constructs have average variance extracted (AVE) values between 0.36 and 0.5, indicating acceptable convergent validity [43].

**Table 5:** Convergent validity of measures of attitude, personality and preference.

**Factor**	**Item**	**SFL**	**CR**	**AVE**
Trust	IAL	0.66	0.80	0.50
	CO	0.66		
	COV	0.74		
	SE	0.76		
Attitude	IE	0.55	0.87	0.37
	RE	0.38		
	AC	0.68		
	FL	0.53		
	PR	0.75		
	AOT	0.74		
	EP	0.55		
Personality	CAO	0.66	0.75	0.36
	HI	0.74		
	OC	0.03		
	AL	0.59		

In this section, we examine a measurement model which includes all the study variables (household income, academic level, safety, comfort, and accessibility) to assess the relationships between latent variables and their indicators. As Table 4 indicates, household income (b = 0.74, p < 0.001), academic level (b = 0.59, p < 0.001), and car ownership (b = 0.66, p < 0.001) are positively related to personality, supporting Hypothesis 1. However, occupation is not significant. For the seven observable variables representing attitudes to mode choice, all factors have significant and positive impacts on attitude. These findings indicate that persons who are well-educated, with high incomes, and have cars in their families tend to be positive about using AVs. With IE, RE, AC, FL, PR, AOT and EP increasing by one unit, attitudes increase by 0.30, 0.15, 0.47, 0.28, 0.56, 0.54, and 0.31, respectively. Of these, predictability and reliability are the main factors. In other words, increasing the predictability and reliability of arrival time would make AVs use more likely. Accessibility is another important service-related attribute which may affect travelers’ decisions to use (or not use) AVs. The above results provide exploratory empirical support for the assumption that attitudes to the services of modes are an important predictor of intention to use AVs in the future.

Regarding the indicators of preferences for AVs, all observable variables have significant and positive impacts on preferences. Safety is the most likely trust preference indicator. Therefore, Hypothesis 2 and Hypothesis 3 are also supported.

#### 4.1.2 Structural model

A structural model was used to determine the goodness of fit statistics of the proposed theoretical structure. As indicated previously, the SEM analysis results obtained an acceptable model fit with the data. In order to evaluate the Goodness of Fit for SEM models, it is suggested to calculate the standardized root mean square residual (SRMR). The SRMR value of the composite factor model in our model was 0.064, showing a good model fit according to the finding that SRMR values close to or less than 0.10 show a good fit [44].

With regard to the factors influencing intention to use AVs, the coefficients shown in Fig 2 for personality and preference have better explanatory power, which also indicates that the PAPI framework is acceptable. Personality has positive impacts on both attitude and preference, significant at the level of 1%. The direct effect represented by the standardized coefficients of personality, attitude, and preference on behavior intention is 0.54, 0.15, and 0.42, respectively, as shown in Table 6. This indicates that, with other conditions unchanged, intention to use AVs rises by 0.54, 0.15, and 0.42 when personality, attitude, and preference increase by one unit. We studied the importance of the service-related attributes of modes in affecting the intention of using AVs. The basic reason for this was to provide an empirical basis for us to make the decision, taking personal attitude as an indirect measure in intention modelling to use AVs. As expected, we observed that personality is the most important factor in the intention to use AVs. The indirect and total effects for the pathway ‘personality-attitude-behavior intention’ are 0.30 and 0.84, as shown in Table 6. Consequently, compared to attitude, changes of social-economic characteristics from personality and preference factors are the major determinants of behavior intention. Moreover, personality also has a positive impact on attitude to mode choice and preference for AVs. The measure for personality illustrates 24% of the variance in the direct measure for preference, 36% of the variance for attitude, and 86% of the variance in future intention to use AVs.

**Table 6:** Standardized direct, indirect and total effects between latent variables.

**Pathways**	**Std. Coeff.**		
	**Direct Effect**	**Indirect Effect**	**Total Effect**
Personality → Attitude	0.60		0.60
Personality → Preference	0.49		0.49
Personality → Intention	0.54	0.30	0.84
Attitude → Intention	0.15		0.15
Preference → Intention	0.42		0.42

Hence, as expected, personality, attitude, and preference are significant predictors of future intention to use AVs, consistent with Hypothesis 4, Hypothesis 5, and Hypothesis 6. On the other hand, the model assumes that personality, attitude, preference and measures cause changes in the latent construct PAPI, implying that changes of indicators lead to changes in the latent construct PAPI.

These results are consistent with the findings of many studies on the relationship between attitude, perceived trust, socioeconomic attributes and intention to use AVs [23, 45]. Related to our present findings, previous studies also show that environmental concern and attitude to AVs play a significant role in estimating decisions on the choice of travel mode. The results of another study show that subjective attitude is the most critical factor affecting travelers’ intention to use AVs [46]. We note that personality (socioeconomic attributes) has a critical effect on intention to use AVs. The insights derived from this study will make a significant contribution to ongoing research related to psychological factors in AV use and are expected to increase AV use by potential customers, such as individuals with high incomes and academic levels.

### 4.2 Discussion and implications

This paper aimed to predict intentions to use an autonomous vehicle. The analysis employed SEM models, as the focus of the study was to identify and explore differences in psychological factors in China when respondents considered using AVs. As shown in the results, perceived trust has a positive impact on behavioral intention, indicating that the important role of trust influences the intention to use AVs. It is clear that individuals will use AVs if they trust AV technology in relation to comfort, safety, convenience and accident responsibility, which confirms the results of Ghazizadeh, Peng [47]. While several studies have found that subjective norms are the weakest indicator of behavior intention [48], some other studies have also confirmed that subjective social pressure is a significant and powerful predictor of intention [49, 50]. The seven items used to measure subjective attitudes in this study also confirmed the conclusion.

In addition, some differences exist across countries about the acceptance or intention to use AVs. A higher percentage of Japanese respondents consider that AV technology is a sensible advance, which may relate to the country increasingly characterized by having a high technology-based economy [51]. Similarly, US participants would prefer autonomous vehicles to general cars [52].

In conclusion, the results of the present study demonstrate that, in keeping with the hypotheses, personality plays the major role in determining the intention to use AVs. Perceived trust in AVs, perceived attitude and preference are also determinants of intention to use AVs. These findings differ somewhat from those of [53]. The above results show that the proposed framework can be applied to understand behavioral intentions to use AVs.

## 5 Limitations

Our paper focuses on users’ intention to use AVs. On the one hand, we discuss the relationship between attitude, personality, preference and behavioral intentions is discussed, and on the other hand the link between personality and attitude and preference is discussed as well. This study could be improved in several aspects to overcome its limitations. The first limitation is that the research samples were comprised of only Chinese survey data. Therefore, future studies could summarize and compare our findings to explore intentions to use AVs in different countries. The second limitation is that this research investigated only the general intention to use AVs and did not include different autonomous modes, such as shared AV, autonomous taxis, and autonomous buses. Thirdly, this study only explored the link between personality, attitude, preference and the intention to use AVs. Other aspects, such as perceived behavioral control (PBC) and subjective norms (SNs), were not investigated. Moreover, future studies could explore the role of attitudes of AV companies, and a more complex structural model could be developed to further study behavioral intentions to use AVs.

## 6 Conclusions and implications

AVs are entering the market, which will have a great impact on future decision making on mode choice in transportation systems. This requires a thorough understanding of travelers’ intentions to use AVs, while little attention has been received to date. In order to understand travelers’ behavioral intention to use AVs, a SEM model was proposed and tested empirically in this paper. The main findings are as follows. AV companies and stakeholders can formulate and implement evidence-based strategies and policies for the improvement of safety, comfort, convenience and accident liability of AVs if they have information on people’s intention to use AVs in the future. However, some people are worried about their safety which will hinder initiatives in AV use.

Based on our findings, we believe that personality is an important factor influencing attitudes to modal services, preference for AVs and intended use of AVs. Preferences and attitudes also have significant direct effects on the intention to use AVs. It will therefore be beneficial for AV operators and policymakers to promote the services of AVs and their policies for different individual groups. Car manufacturers and governments can promote the benefits of AV technology (such as improving mobility and easing traffic congestion) to increase the willingness of travelers to choose AVs. Therefore, taking into account passengers’ evaluation of AVs (positive / negative), AV companies and decision makers can develop more specific measures to improve the perception of AVs. Note that different from previous studies [46], the present study indicates that attitudes to modal services contribute the least effect on intention to use AVs. Our findings indicate that the respondents do not care so much about whether they can rest in the vehicle, which contributes half to the predictability and reliability. The intention to use AVs may be more related to concerns about safety and technical failures [22, 54] than to the feared loss of driving pleasure or unwillingness to relinquish control [55]. As a result, we suggest that in order to promote the intention in AVs use, relevant organizations should focus on improving travelers’ trust of AVs to reduce the perceived risks.

These results have implications for policy and practice. Knowledge of passengers’ perceptions and intentions to use AVs is important for finding feasible interventions to guide and satisfy passengers. As described in previous studies, passengers’ behavioral intentions indicate whether they will use a new service [6, 15, 56]. Our findings can be used to formulate effective measures with the aim of improving AV safety and individuals’ knowledge about AVs. According to our findings, another area where possible interventions and policies may be adopted is how AV companies are organized and operated. Relevant government departments should issue AV safety regulations and standards, and official regulation is needed if safety standards need to be improved.

The abbreviations used in the manuscript and their meanings are listed as follows:

## Supporting information

S1 File (SAV)

## Abbreviations

AVs: autonomous vehicles
SEM: structural equation modelling
CFA: confirmatory factor analysis
CFI: Comparative Fit Index
GFI: Goodness of Fit Index
IFI: Incremental Fit Index
RMSEA: Root Mean Square Error of Approximation
TLI: Tucker-Lewis Index
IE: interior environment
RE: rest
AC: accessibility
FL: flexibility
PR: predictability
AOT: reliability
EP: environmental preference
SE: safety
CO: comfort
COV: convenience
IAL: responsibility
PAPI: personality-attitude & preference-intention
SFL: standardized factor loading
AVE: average variance extracted
CR: construct reliability
PBC: perceived behavioral control
SN: subjective norm
